# Association of an *APBA3* Missense Variant with Risk of Premature Ovarian Failure in the Korean Female Population

**DOI:** 10.3390/jpm10040193

**Published:** 2020-10-26

**Authors:** JeongMan Park, YoungJoon Park, Insong Koh, Nam Keun Kim, Kwang-Hyun Baek, Bo-Seong Yun, Kyung Ju Lee, Jae Yun Song, Eunil Lee, KyuBum Kwack

**Affiliations:** 1 Institute Department of Biomedical Science, College of Life Science, CHA University, Seongnam, Gyeonggi-do 13488, Korea; jungman.park@chauniv.ac.kr (J.P.); yjparkcb@gmail.com (Y.P.); nkkim@cha.ac.kr (N.K.K.); baek@cha.ac.kr (K.-H.B.); 2 Department of Biomedical Informatics, Hanyang University, Seoul 04763, Korea; insong@hanyang.ac.kr; 3 Department of Obstetrics and Gynecology, CHA Gangnam Medical Center, CHA University, Seongnam, Gyeonggi-do 13497, Korea; bosungyun@chamc.co.kr; 4 Department of Obstetrics and Gynecology, Korea University Medical Center, Seoul 02841, Korea; drlkj52551@korea.ac.kr; 5 Department of Preventive Medicine, College of Medicine, Korea University, Seoul 02841, Korea; sjyuni105@gmail.com (J.Y.S.); eunil@korea.ac.kr (E.L.)

**Keywords:** follicle stimulating hormone, primary ovarian failure, genome-wide association study, hypoxia-inducible factor 1, ovarian follicle, cell proliferation, autophagy

## Abstract

Premature ovarian failure (POF) is a complex disease of which the etiology is influenced by numerous genetic variations. Several POF candidate genes have been reported. However, no causal genes with high odds ratio (OR) have yet been discovered. This study included 564 females of Korean ethnicity, comprising 60 patients with POF and 182 controls in the discovery set and 105 patients with POF and 217 controls in the replication set. We conducted genome-wide association analysis to search for novel candidate genes predicted to influence POF development using Axiom Precision Medicine Research Arrays and additive model logistic regression analysis. One statistically significant single nucleotide polymorphism (SNP), rs55941146, which encodes a missense alteration (Val > Gly) in the *APBA3* gene, was identified with OR values for association with POF of 13.33 and 4.628 in the discovery and replication sets, respectively. No rs55941146 minor allele homozygotes were present in either cases or controls. The APBA3 protein binds FIH-1 that inhibits hypoxia inducible factor-1α (HIF-1α). HIF-1α contributes to granulosa cell proliferation, which is crucial for ovarian follicle growth, by regulating cell proliferation factors and follicle stimulating hormone-mediated autophagy. Our data demonstrate that *APBA3* is a candidate novel causal gene for POF.

## 1. Introduction

Premature ovarian failure (POF) is an idiopathic, complex disease in which menopause occurs before the age of 40 years [1]. The exact cause of POF remains unknown. However, many factors may contribute to its development, including autoimmune disease, radiation therapy, anti-cancer drugs, chromosomal abnormalities, and mental status [2,3,4]. The diagnostic criteria for POF in premenopausal females include an increase of serum follicle stimulating hormone (FSH) levels (>40 mIU/mL), measured twice in the same month, alongside the presence of amenorrhea for 6 months before the normal age of menopause onset [5]. Without appropriate hormone replacement therapy, women with POF can develop severe health issues, including not only failure of normal ovary function, but also various other problems, including cardiovascular disease, coronary artery disease, and stroke [6,7].

The ovarian follicle is important to oocyte growth and folliculogenesis is a crucial mechanism contributing to female fertility [8,9,10]. There are four stages in folliculogenesis. In the primordial stage, small, dormant primordial follicles are enclosed by a single layer of granulosa cells (GCs) in the ovarian cortex [8,9]. In the primary stage, oocytes and GCs exhibit dramatic growth, and meanwhile, GCs change shape from a flat to cuboid form [8,9]. In the secondary stage, stroma-like theca cells enclose the follicles, and GCs increase until there are nine layers [8,9]. In the tertiary and preovulatory stage, the antrum is fully formed in an FSH dependent manner, and all follicles, except for one, undergo follicular atresia [11,12,13]. GCs fulfill their important roles in the ovarian follicle by interacting with oocytes via gap junctions to provide nutrients and signaling molecules [14]. GCs are also important during follicle development under the influence of FSH and luteinizing hormone, which induce GC proliferation [13,15,16]. Furthermore, FSH promotes the development of preantral follicles and induces an anti-apoptotic process in antral follicles [15,16,17,18].

There have been numerous reports of associations between single nucleotide polymorphisms (SNPs) and POF onset [19,20,21]. Therefore, we tried to find candidates in rare variants using the precision medicine research array (PMRA) chip, which contains low allele frequency markers. By conducting a genome-wide association study (GWAS) to identify novel variants associated with POF in Korean women, we detected an SNP variant that could substantially increase the risk of POF occurrence.

## 2. Results

### 2.1. Patients and Kinship Analysis

Subjects comprised a total of 564 females of Korean ethnicity. Sixty patients with POF and 182 controls in the discovery set, and 120 patients with POF and 218 controls in the replication set. Patient genomic DNA samples were genotyped using an PMRA. Kinship analysis detected 30,876 associations among 242 individuals in the discovery set, and there were no first and second degree relationships. Kinship analysis was also conducted in the replication set and a total of 322 individuals were left. Our results indicated that none of the study participants were related.

### 2.2. GWAS

Raw data generated by genotyping of the discovery set using Axiom PMRA chips were first filtered by removing markers with missing annotation and deletion/insertion markers (*n* = 346,668). All individuals passed the individual-level missingness threshold of <0.1. Application of Hardy-Weinberg equilibrium (HWE) threshold of >1.0 × 10^−5^ to the control group resulted in the exclusion of 7937 markers. A marker-level missingness threshold of >0.001 excluded 210,158 markers because of low genotyping rates, and the minor allele frequency threshold (<0.05) further excluded 255,378 markers. Finally, only autosomal markers were included in our analysis. Hence, 20,046 markers mapped to the X and Y chromosomes or mitochondrial DNA were excluded. Consequently, a total of 625,170 markers were removed, while 277,022 remained. After additive model association logistic regression analysis, 45 SNPs were identified as significantly associated with POF in the discovery set. An identical analysis was then conducted using the replication set, resulting in validation of only one of the 45 SNPs from the discovery set with a significant *p*-value (2.996 × 10^−7^) followed by a Bonferroni correction (Table 1). This significant SNP, rs55941146, maps to the *APBA3* coding region (A > C) on chromosome 19.

**Figure 1:**
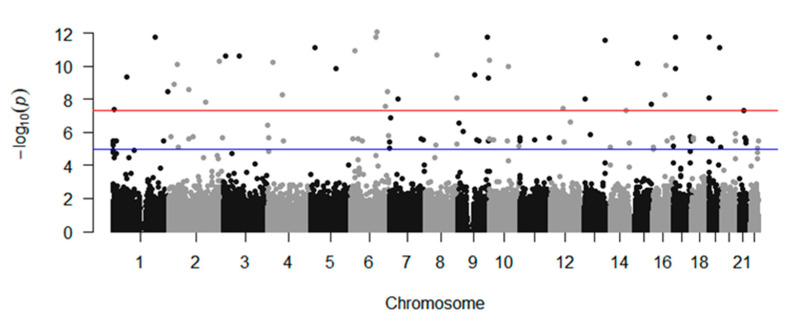
Manhattan plot for genome wide association study (GWAS) data from the Korean female population, showing −log10 (*p*-values) from GWAS and imputation analysis plotted against the chromosome position. Each color represents a different chromosome. The lower line indicates the suggested association threshold (*p* = 1.0 × 10^−5^) while the upper line indicates the genome-wide significance threshold (*p* = 5.0 × 10^−8^).

**Figure 2:**
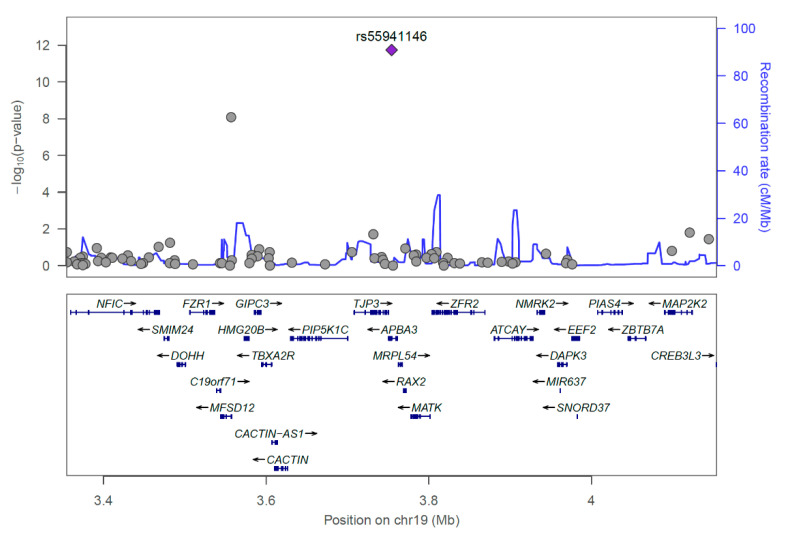
Regional association plot for loci significantly associated with premature ovarian failure in the Korean female population: chr19:3354338−4154338 [3354338−4154338] (*APBA3*).

In the discovery set, significant data were visualized with Manhattan and quantile-quantile plots (Figure 1, Supplementary Figure S1). Furthermore, the recombination rate of rs55941146 with other SNPs in a range of ±400 kb was analyzed using LocusZoom (Figure 2). Additive model analysis of rs55941146 and POF association, generated odds ratio (OR) values of 13.33 and 4.628 in the discovery and validation sets, respectively (Table 1). The entire process of quality control (QC), applied threshold in each process, and the number of excluded markers are included in the flow chart (Supplementary Figure S2). The minor allele of rs55941146 was only present in the heterozygous form in both patients and controls (Supplementary Table S1). The minor allele frequencies of the associated SNP in other populations, according to 1000 genomes data, was compared with that in the POF group (Supplementary Table S2). The frequency of variation of rs55941146 is 0 in the East Asian group, but 0.4 in Korean POF patients.

### 2.3. Predicted Influence of rs55941146 on Protein Structure

The rs55941146 variant is a missense SNP (A > C) in the *APBA3* coding region, which causes a valine to glycine substitution at residue 206 in the 575 amino acid full-length APBA3 protein. Scores (0.01 and 0.999) generated using the Sorting Intolerant From Tolerant (SIFT) and PolyPhen-2 programs, respectively, indicated that the rs55941146 variant is predicted to have a deleterious effect on the APBA3 amino acid sequence, and is likely damaging to the APBA3 three-dimensional structure.

## 3. Discussion

The *APBA3* gene encodes amyloid-beta precursor protein binding family A member 3, which is also referred to as mammalian uncoordinated 18-1 (MUNC 18-1) interacting protein 3 (MINT3). APBA3 functions both to modulate processing of the amyloid-beta precursor protein (APP) by binding to its C-terminal domain and regulating factor-inhibiting hypoxia inducible factor-1 (FIH-1) via its N-terminal domain [22,23]. FIH-1 inhibits hypoxia inducible factor-1 (HIF-1), which regulates glucose metabolism under hypoxic conditions [24]. FIH-1 is an asparaginyl hydroxylase enzyme that promotes asparaginyl hydroxylation of the COOH-terminal transactivation domain (CAD) of HIF-1, thereby reducing HIF-1 function [25,26]. Both APBA3 and HIF-1α contain identical domains that compete for binding to FIH-1 [22]. Hence, if APBA3 is bound to FIH-1, the asparaginyl hydroxylase modification of the HIF-1 CAD region mediated by FIH-1 is inhibited [22]. Therefore, inhibition of FIH-1 by APBA3 leads to increased HIF-1 expression. In contrast, if HIF-1α binds to FIH-1, the asparagine in the CAD region of HIF-1α is modified [27], leading to the degradation of HIF-1α by the ubiquitin-proteasome pathway [28]. Under normal conditions, FIH-1 interacts with HIF-1α leading to the HIF-1 degradation, while, during hypoxia, HIF-1α is stabilized and activated due to the interaction of FIH-1 with APBA3 [28].

Gonadotrophins, including FSH, have established roles in stimulating follicle growth and preventing the GC apoptosis associated with follicle atresia [29,30,31]. There is ample evidence supporting the importance of HIF-1α in angiogenesis, cell proliferation, and metabolic conversion from oxidative phosphorylation to glycolysis [32,33,34,35,36]. Inevitably, various stresses, including hypoxia and nutritional stress, occur during follicle growth, which involves intense cell proliferation [36]. During ovarian follicle growth, cell proliferation is promoted by FSH, which stimulates accumulation of HIF-1, and HIF-1α increases in response to treatment with FSH both in vitro and in vivo [29,37]. Hence, HIF-1α is a factor downstream of FSH [38], and FSH also stimulates HIF-1α transcription and translation in ovarian cancer cells [39].

Ovarian follicle atresia, in which immature follicles degenerate, is an important stage of follicle growth [40], triggered by GC apoptosis [41]. The enhancement of autophagy stimulates GC apoptosis [42] and is induced by conditions occurring in primordial follicles, including starvation [43]. The absence of autophagy leads to the accumulation of aging-related catabolic waste products during folliculogenesis [29,44]. Hence, autophagy protects ovarian follicles, and, specifically, oocytes, from abnormal conditions, including starvation, which occur in primordial follicles [43]. In summary, appropriate FSH-mediated autophagy, which is important for removing waste and maintaining metabolic balance, is necessary for ovarian follicle growth and preservation of primordial follicles [29,45].

HIF-1α is important, not only as a factor downstream of FSH-mediated autophagy, but also an inducer of proliferation factors. Knockdown of HIF-1α induces downregulation of proliferation markers, such as cyclin D2 (CCND2) and proliferating cell nuclear antigen (PCNA) [46]. Furthermore, HIF-1 regulates cell proliferation differentially in hypoxia and normoxia [46]. PCNA and CCND2 are proliferation markers in various tissues, including ovarian follicles [47,48], and both markers are used to assess GC proliferation levels both in vitro and in vivo [46,49,50,51]. During ovarian follicle growth, HIF-1α influences the expression levels of numerous factors, including PCNA and CCND2 [48].

The majority of candidate genes identified by GWAS as associated with various diseases have OR values < 1.5, and such genes with relatively low OR values have a weak impact in increasing disease risk [52]. Compared with the majority of reported GWAS findings, our resulting OR was remarkably high. Consequently, we infer that rs55941146 likely has a substantial influence on POF development.

In conclusion, we identified a variant with a high OR for association with POF, relative to previously reported candidate genes, which is predicted to have a detrimental impact on the amino acid sequence and tertiary structure of the APBA3 protein. This *APBA3* SNP (rs55941146) may influence FSH-mediated autophagy and transcription factor induction by association with HIF-1α in granulosa cells. This would be expected to induce down-regulation of autophagy, and of various transcription factors in pre-antral and antral follicles stages, under hypoxic conditions. Variation in APBA3, which regulates the FIH-1/HIF-1 pathway, may lead to impaired FIH-1 downregulation during hypoxia and consequent inappropriate inactivation of HIF-1. Reduction in the HIF-1α level can lead to suppression of normal levels of autophagy and increased transcriptional activity. Therefore, our data suggest that *APBA3* is associated with POF in the Korean female population and represents a new candidate gene for this condition.

## 4. Materials and Methods

### 4.1. Patient Recruitment

For the discovery set, 242 women were, retrospectively, selected for inclusion in this study from a total of 367 individuals who visited Korea University Anam Medical Center from 2016 to 2019 and Bundang CHA Hospital until 2010. For the replication set, 322 women were, retrospectively, selected for inclusion in the analysis from 338 individuals who gathered by CHA University until 2004. Samples used in this GWAS study was authorized by the Institutional Review Board of Korea University Anam Medical Center (2016AN0216) and the Institutional Review Board of Bundang CHA Hospital (2010123). The samples used in the replication study were authorized by the Institutional Review Board of CHA University (1044308-201310-BR-002-01).

### 4.2. Whole Genome Genotyping Using the PMRA Chip

DNA was extracted from peripheral blood samples from the 580 individuals recruited to the study. SNP array analysis was conducted using Axiom Precision Medicine Research Array (PMRA) 2.0 chips. SNPs (*n* = 902,380) were initially evaluated in samples from the 242 individuals in the discovery set. SNP data pre-processing was performed using the Affymetrix Power Tool to generate dish quality control (DQC) values to ensure samples were of sufficient quality for analysis. The dish quality control (DQC) threshold value was 0.82, where samples with DQC values < 0.82 were excluded from further genotype analysis. Quality control of the remaining samples was by sex check to identify differences between clinically determined sex and sex predicted based on genotype data. In the following step, samples with call rate values less than the threshold (97%) were removed. Finally, abnormal rates of heterozygosity and plate quality control values were evaluated and relationship tests were performed.

### 4.3. GWAS Process

Korean patients with POF (*n* = 60) and controls (*n* = 182) in the discovery set, and patients (*n* = 105) and controls (*n* = 217) in the replication set, were included, comprising a total of 564 samples. Data were analyzed using descriptive statistics and additive model logistic regression analysis. For the additive model association analysis, thresholds were as follows: individual-level missingness <0.1, marker-level missingness <0.001, and minor allele frequency >0.05. HWE was applied only in controls, using an empirical *p*-value threshold of >1.0 × 10^−5^. Finally, 166,866 SNPs were included in the analysis.

### 4.4. Statistical Analysis

To account for multiple comparisons, *p*-values were corrected using the Bonferroni method by applying the formula, α = 0.05/N. SNP data analysis was conducted using PLINK v1.07 (Free Software Foundation, Inc. Boston, MA, USA) [53], LocusZoom v0.12 (Department of Biostatistics and Center for Statistical Genetics, University of Michigan, Ann Arbor, MI, USA) [54], and qqman (a package in R, A Language and Environment for Statistical Computing, R Core Team, R Foundation for Statistical Computing, Vienna, Austria [55]. Kinship analysis was conducted using Kinship-based Inference for Genome-wide association studies (KING) [56].

### 4.5. Protein Structure Analysis

SIFT v6.2.1 (https://sift.bii.a-star.edu.sg/, 180820) was used to analyze the functional consequences of the SNPs identified and protein biological function and PolyPhen-2 v2.2.2. (http://genetics.bwh.harvard.edu/pph2, 180820) was used to predict effects of variants on both the amino acid sequence and protein tertiary structure.

## Figures and Tables

**Table 1:** The statistical results of additive model logistic regression analysis in the discovery and replication sets. CHR, chromosome. SNP, single nucleotide polymorphism. BP, physical position. A1, minor allele. TEST, logistic regression test method for the genetic model. OR, odds ratio. STAT, Coefficient t-statistic. P, *p*-value.

Information					Discovery			Replication		
CHR	SNP	BP	A1	TEST	OR	STAT	*p*	OR	STAT	*p*
1	rs3007782	4,472,736	T	ADD	4.287	5.488	4.06 × 10^−8^	1.061	0.1557	0.8763
1	rs74918369	61,457,396	C	ADD	8.071	6.242	4.31 × 10^−10^	2.133	1.283	0.1994
1	rs115143460	1.9 × 10^8^	A	ADD	13.33	7.047	1.83 × 10^−12^	3.37 × 10^−9^	0.0009	0.9993
1	rs12026894	2.45 × 10^8^	A	ADD	6.483	5.903	3.57 × 10^−9^	1.33	1.01	0.3125
2	rs12615054	22,223,766	G	ADD	7.526	6.069	1.29 × 10^−9^	0.9204	−0.2382	0.8117
2	rs141292341	37,310,903	T	ADD	10.14	6.5	8.05 × 10^−11^	1.38	0.4903	0.6239
2	rs145263938	87,842,313	T	ADD	5.618	5.956	2.58 × 10^−9^	0.5837	−1.115	0.2648
2	rs12692712	1.65 × 10^8^	C	ADD	4.92	5.649	1.61 × 10^−8^	1.668	1.819	0.06891
2	rs79371157	2.28 × 10^8^	T	ADD	9.601	6.56	5.38 × 10^−11^	0.812	−0.3447	0.7303
3	rs28630998	13,245,725	G	ADD	8.74	6.678	2.42 × 10^−11^	4.312	3.007	0.00264
3	rs2323277	74,110,248	A	ADD	11.5	6.684	2.33 × 10^−11^	1.209	0.4123	0.6801
4	rs141002338	24,750,774	C	ADD	10.45	6.536	6.32 × 10^−11^	0.5075	−1.032	0.3019
4	rs1510746	67,469,201	C	ADD	6.439	5.839	5.25 × 10^−9^	0.6251	−0.9697	0.3322
5	rs144522971	22,316,095	T	ADD	11.99	6.853	7.22 × 10^−12^	2.813	1.338	0.1811
5	rs296486	1.13 × 10^8^	A	ADD	8.526	6.41	1.45 × 10^−10^	2.628	2.506	0.01222
6	rs117716146	16,032,432	T	ADD	11.65	6.789	1.13 × 10^−11^	0.8826	−0.1782	0.8585
6	rs111721931	1.1 × 10^8^	G	ADD	13.21	7.047	1.83 × 10^−12^	1.26 × 10^−9^	−0.0019	0.9985
6	rs137945470	1.18 × 10^8^	A	ADD	14.85	7.153	8.51 × 10^−13^	2.078	0.5152	0.6064
6	rs11759078	1.53 × 10^8^	G	ADD	4.917	5.565	2.62 × 10^−8^	0.9774	−0.0669	0.9467
6	rs113416075	1.66 × 10^8^	A	ADD	7.197	5.911	3.39 × 10^−9^	0.577	−1.466	0.1428
7	rs11761631	6,932,435	A	ADD	3.595	5.276	1.32 × 10^−7^	0.9426	−0.2598	0.795
7	rs3807170	37,898,032	T	ADD	6.065	5.735	9.76 × 10^−9^	0.721	−0.7404	0.4591
8	rs140245000	55,592,196	A	ADD	10.09	6.695	2.16 × 10^−11^	2.11	1.041	0.298
8	rs137854443	1.45 × 10^8^	C	ADD	0.208	−5.769	7.99 × 10^−9^	NA	NA	NA
9	rs77096227	8,010,136	C	ADD	3.712	5.139	2.76 × 10^−7^	0.9232	−0.3035	0.7615
9	rs149748677	78,256,026	A	ADD	8.312	6.272	3.57 × 10^−10^	0.5173	−0.5861	0.5578
9	rs146186513	1.31 × 10^8^	G	ADD	13.33	7.047	1.83 × 10^−12^	NA	NA	NA
9	rs77609276	1.38 × 10^8^	T	ADD	8.534	6.201	5.60 × 10^−10^	0.7848	−0.4485	0.6538
10	rs78014704	1,842,302	T	ADD	9.568	6.59	4.39 × 10^−11^	0.5369	−1.34	0.1803
10	rs34498403	86,799,865	T	ADD	8.2	6.455	1.09 × 10^−10^	1.373	0.3439	0.7309
12	rs10877123	58,881,273	A	ADD	3.971	5.498	3.84 × 10^−8^	1.111	0.4515	0.6516
12	rs74650809	92,513,269	A	ADD	3.578	5.176	2.27 × 10^−7^	1.05	0.2121	0.8321
13	rs56238336	23,029,868	C	ADD	4.942	5.737	9.65 × 10^−9^	1.546	1.448	0.1476
13	rs146581261	1.11 × 10^8^	A	ADD	14.3	6.992	2.71 × 10^−12^	1.682	0.9786	0.3278
14	rs1957293	92,708,417	A	ADD	4.209	5.448	5.10 × 10^−8^	1.068	0.2128	0.8315
15	rs78879506	37,133,840	A	ADD	10.6	6.534	6.39 × 10^−11^	1.125	0.3918	0.6952
15	rs57049930	93,855,283	T	ADD	5.072	5.615	1.97 × 10^−8^	0.9163	−0.2447	0.8067
16	rs77141302	54,570,512	C	ADD	4.301	5.826	5.68 × 10^−9^	1.138	0.4584	0.6466
16	rs149428	58,668,566	A	ADD	8.74	6.479	9.21 × 10^−11^	0.2768	−1.683	0.09247
17	rs146234219	9,038,490	T	ADD	13.33	7.047	1.83 × 10^−12^	3.36 × 10^−9^	0.0009	0.9993
17	rs17773918	11,399,739	G	ADD	8.535	6.425	1.32 × 10^−10^	3.569	1.718	0.08571
19	rs1715092	3,557,005	A	ADD	4.858	5.762	8.31 × 10^−9^	0.9295	−0.2799	0.7796
19	rs55941146	3,754,338	C	ADD	13.33	7.047	1.83 × 10^−12^	4.75	3.885	0.000102
19	rs918371	48,808,545	T	ADD	11.69	6.854	7.17 × 10^−12^	1.29 × 10^−9^	−0.0008	0.9993
21	rs16991683	35,800,134	A	ADD	4.26	5.47	4.49 × 10^−8^	0.7194	−1.104	0.2696

## Supplementary Materials

The following are available online at https://www.mdpi.com/2075-4426/10/4/193/s1, Figure S1. Quantile-quantile plot for GWAS data from 60 patients with premature ovarian failure and 182 controls. Figure S2. Flow chart of data processing in the discovery and replication sets. Table S1. Genotypes of rs55941146 in samples included in this study. Table S2. Comparison of allele frequency in recruited samples and variable population of 1000 genomes. EAS, east Asian; CDX, Chinese Dai in Xishuangbanna, China; CHB, Han Chinese in Beijing, China; CHS, Southern Han Chinese; JPT, Japanese in Tokyo, Japan; KHV, Kinh in Ho Chi Minh City, Vietnam.
