# Supplementary material for: Association of an APBA3 Missense Variant with Risk of Premature Ovarian Failure in the Korean Female Population
Source: J Pers Med. 2020 Oct 26;10(4):193. doi: 10.3390/jpm10040193 (PMC7720130; doi:10.3390/jpm10040193)
Supplement: Supplementary file 1 [file jpm-10-00193-s001.zip › supplimentary.docx]

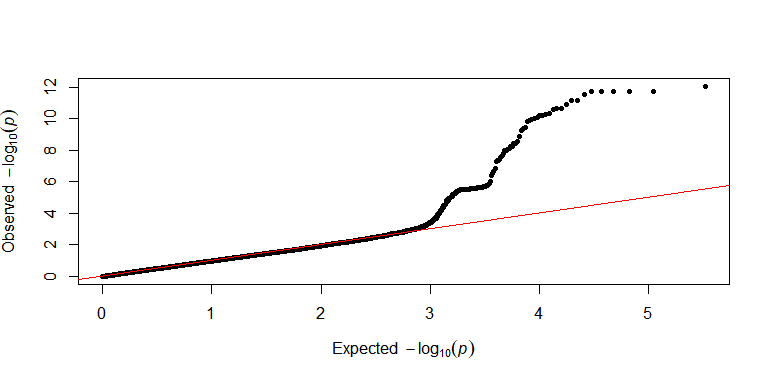


Figure S1. Quantile-quantile plot for GWAS data from 60 patients with premature ovarian failure and 182 controls.


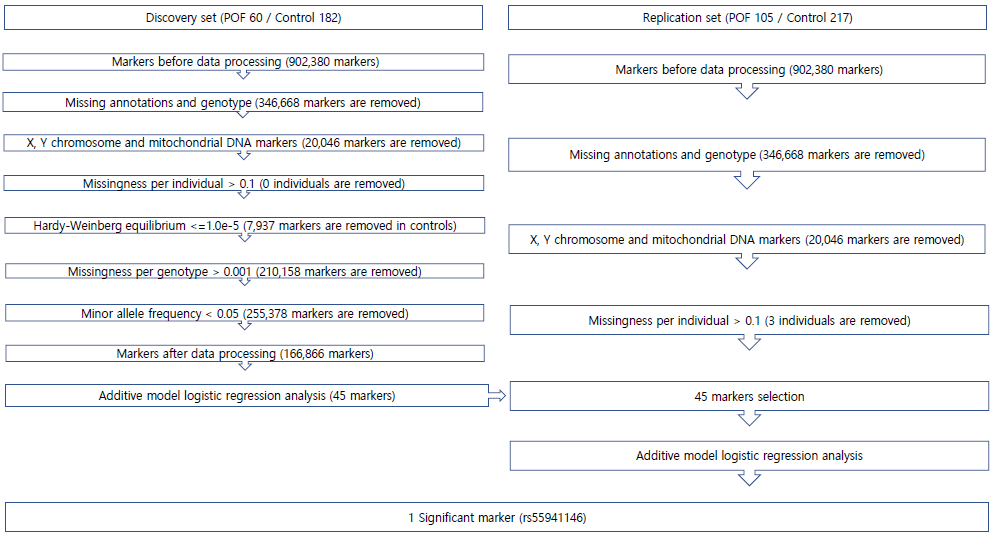


Figure S2. Flow chart of data processing in the discovery and replication sets.

|  | GWAS | | Replication | |
| --- | --- | --- | --- | --- |
| Genotype | Case (n) | Control (n) | Case (n) | Control (n) |
| AA | 12 | 140 | 60 | 190 |
| AC | 48 | 42 | 18 | 12 |
| CC | 0 | 0 | 0 | 0 |

Table S1. Genotypes of rs55941146 in samples included in this study

Table S2. Comparison of allele frequency in recruited samples and variable population of 1000 genomes. EAS, east Asian; CDX, Chinese Dai in Xishuangbanna, China; CHB, Han Chinese in Beijing, China; CHS, Southern Han Chinese; JPT, Japanese in Tokyo, Japan; KHV, Kinh in Ho Chi Minh City, Vietnam.

| Population (EAS) | Allele frequency (n) | Genotype frequency (n) |
| --- | --- | --- |
| EAS | A: 1.000 (1008) \| C: 0 (0) | A\|A: 1.000 (504) |
| CDX | A: 1.000 (186) \| C: 0 (0) | A\|A: 1.000 (93) |
| CHB | A: 1.000 (206) \| C: 0 (0) | A\|A: 1.000 (103) |
| CHS | A: 1.000 (210) \| C: 0 (0) | A\|A: 1.000 (105) |
| JPT | A: 1.000 (208) \| C: 0 (0) | A\|A: 1.000 (104) |
| KHV | A: 1.000 (198) \| C: 0 (0) | A\|A: 1.000 (99) |
| POF patients | A: 0.600 (72) \| C: 0.400 (48) | A\|A: 0.200 (12) A\|C: 0.800 (48) |
